# Explaining the Role of Proximate Determinants on Fertility Decline among Poor and Non-Poor in Asian Countries

**DOI:** 10.1371/journal.pone.0115441

**Published:** 2015-02-17

**Authors:** Nabanita Majumder, Faujdar Ram

**Affiliations:** International Institute for Population Sciences, Mumbai, India; Texas Tech University Health Science Centers, UNITED STATES

## Abstract

**Objectives:**

We examined the overall contributions of the poor and non-poor in fertility decline across the Asian countries. Further, we analyzed the direct and indirect factors that determine the reproductive behaviour of two distinct population sub-groups.

**Design:**

Data from several new rounds of DHS surveys are available over the past few years. The DHS provides cross-nationally comparable and useful data on fertility, family planning, maternal and child health along with the other information. Six selected Asian countries namely: Bangladesh, India, Indonesia, Nepal, Philippines, and Vietnam are considered for the purpose of the study. Three rounds of DHS surveys for each country (except Vietnam) are considered in the present study.

**Methods:**

Economic status is measured by computing a “wealth index”, i.e. a composite indicator constructed by aggregating data on asset ownership and housing characteristics using principal components analysis (PCA). Computed household wealth index has been broken into three equal parts (33.3 percent each) and the lowest and the highest 33.3 percent is considered as poor and non-poor respectively. The Bongaarts model was employed to quantify the contribution of each of the proximate determinants of fertility among poor and non-poor women.

**Results:**

Fertility reduction across all population subgroups is now an established fact despite the diversity in the level of socio-economic development in Asian countries. It is clear from the analysis that fertility has declined irrespective of economic status at varying degrees within and across the countries which can be attributed to the increasing level of contraceptive use especially among poor women. Over the period of time changing marriage pattern and induced abortion are playing an important role in reducing fertility among poor women.

**Conclusions:**

Fertility decline among majority of the poor women across the Asian countries is accompanied by high prevalence of contraceptive use followed by changing marriage pattern and induced abortion.

## Introduction

Fertility decline is strongly associated with socio-economic development since changed or improved socio-economic conditions motivate individuals, couples and families to reduce fertility [1, 2, 3, 4, 5]. During the 1990s much of the world experienced substantial economic growth. In addition, increase in child survival rate reduces the demand for large families as ‘insurance’ against infant and child mortality, and increasing demand for human capital (education) prolongs the period of child dependence and thus the cost of children incurred by parents/families. Finally, women’s greater human capital and opportunity for female employment increases women’s status and autonomy and the opportunity costs of childbearing.

Contrary to common wisdom, the worldwide fertility decline which occurred over the past forty years in developing countries was anticipated by demographers and population forecasters. What was not really anticipated was its magnitude. Since the transition from high to low fertility is now virtually universal, it is clear that its onset does not depend on the level of development and that the path it will follow is not necessarily determined by socio-economic factors such as levels of education, female employment, or urbanization [6, 1, 7]. Therefore, proximate determinants of fertility are more relevant in explaining changing fertility behavior since they represent the mechanisms through which the reduction of fertility has occurred. Therefore, it is imperative to study the proximate determinants of fertility to assess the likelihood of fertility reduction in countries with intermediate fertility levels at present that will reach replacement level in near future. In addition, changes and contribution of proximate determinants reveal the turn down factor of fertility precisely.

A number of experiences confirm that the initial fertility reduction was instigated by educated and socio-economically affluent group of women [8, 3, 9, 10]. However in past three to four decades prominent changes in reproductive behaviour have been documented. Literature confirmed that the bulk of fertility decline is now occurring in most of the regions of the developing world among women without education and this transition is being driven in a major way by the increasing contraceptive prevalence rates among illiterate or less educated women [11, 12, 13]. The substantial decline that took place in few poor and largely agricultural countries such as Bangladesh was completely unexpected and is forcing a major revision of theories about fertility decline [6]. Since the fertility level was being controlled by the higher socio-economic group initially, the later decline in its level may be the response of the fertility control among the destitute group in the society. As poor women join in the above transition process (which has not yet happened in many societies that are in mid-transition), poverty and inequality reduction affects increases [14].

During the two decades, Asian fertility declined dramatically [15]. Almost all the Asian countries have experienced fertility decline in last two decades though, at various levels. The onset of the fertility transition from high to low fertility rates was clearly a widespread phenomenon in Asia during that period. In particular, majority of the fertility decline in Asia have occurred among poor, with large rural and illiterate strata [1] in most populous countries like Bangladesh, India, China, Indonesia, Sri Lanka and Vietnam. Together their populations’ amount is 76 percent of the population of Asia and 60 percent of the population of all developing countries. Interestingly, demographers and policy makers did not expect such rapid fertility decline among rural, poor and illiterate section of the society [16]. Therefore, it is imperative to explore the fertility behavior and the direct and indirect factors shaping the child bearing process among economically indigent section of the society. In addition, their contribution in the recent ongoing fertility transition which will highlight their needs and preparedness for a sustainable contribution in fertility reduction.

## Review of Literature

The recent decline in fertility in developing countries created interest among researchers, policy makers and academicians. This is because such a dramatic change in fertility has occurred without a substantial improvement in socio-economic status, health conditions and outlier factors thought to be needed to bring about a fertility decline. Some argue that the decline in the fertility level was achieved mainly because of a successful family planning programme [17]. Population development programmes have, no doubt, contributed to the fertility decline. However, several biological, behavioural and cultural factors are also involved. Bongaarts (1978) termed these factors as proximate determinants of fertility, since they directly, affect fertility; all other social, economic and environmental factors affect fertility through these variables [18].

### Age at Marriage

Marriage, the legal and cultural institution that sanctions childbearing, is one of the most important determinants of fertility. Age at first marriage has a major effect on childbearing because women who marry early have, on average, a longer period of exposure to the risk of becoming pregnant and a greater number of lifetime births. It is widely acknowledged that age at marriage has a significant influence on fertility, particularly in countries where childbearing occurs within marriage. Therefore, in societies where child bearing prior to marriage is not socially acceptable, postponement of marriage contributes significantly towards a reduction in the level of fertility by shortening the total reproductive span of women. This in turn reduces the number of children a woman is likely to have and has a negative impact on the population growth rate of a country. Age pattern of marriage was discovered as an important factor for reducing marital fertility rate due to late marriage and a higher proportion of celibacy in developed countries [19, 20, 21, 22, 9]. Western Europe responded to the Malthusian challenge of over population in the nineteenth century with an increase in the marriage age, which significantly cut fertility. In the developing countries, some drop in fertility is occurring because of a rise in the marriage age due to increasing education and employment, and a legal attempt through legislation to prohibit early marriages [23]. In few developing countries in Asia and Africa, where the use of effective contraceptive methods are low, delayed marriage has played an important role to engrave the fertility level [24].

A review of the recent literature provided evidence that, despite the changing pattern towards later marriage, Asian countries display considerable variations in marriage patterns. Interestingly, one pattern is shared in all Asian countries: 95 per cent or more of women ultimately marry [25]. Social and economic forces are transforming traditional marriage pattern in Asia. In the near future marriage behavior in Asia will reflect the paths of key modernization process: educational development, urbanization, and the expansion of non-agricultural employment. It is quite likely that each of these processes will encourage continued marriage delay and perhaps even the greater prevalence of celibacy in the coming decades [23].

An increasing trend in age at first marriage was found in Vietnam [26]. The increase was modest but significant over time after controlling for other socioeconomic variables. Education, type of residence, wealth, age, region, and ethnicity were strongly related to age at first marriage. Study also revealed that there were significant proportion of women who got married during adolescent especially among rural; minority; and less educated women. Another study [27] found that education and age at first marriage are strongly associated both at the individual level and at the societal level: a woman who has attended secondary school is considerably less likely to marry during adolescence, and with higher proportion of women with a secondary education, the proportion of women who marry as adolescents is lower.

Marriage change has played a considerable role in the recent fertility declines in a number of Asian countries [28]. Both delay in marriage and postponement of childbearing by married couples held down fertility. Among the many factors lowering desired childbearing among married couples in Pacific Asia, prominent possibilities are uncertainty of continued employment, conflict of work and family responsibilities, lack of appropriate policies to support child-rearing, the ideology of the “quality” child, financial costs of child-rearing, gender ideologies on housework, and difficulties of the urban environment. Possibly rising individualism also plays a part.

Unlike in Western countries where marriage is not a pre-condition for child bearing, in most Asian countries child bearing prior to marriage is not socially acceptable and is therefore extremely uncommon. Postponement of marriage therefore contributes significantly towards a reduction in the level of fertility by shortening the total reproductive span of women, which in turn reduces the number of children a woman is likely to have and hence reduces the population growth rate of a country. The age at marriage indeed has a significant effect on the fertility of women—an increase in the age at marriage significantly reduces total fertility of women. Further, the higher the education level of the woman the stronger is the effect of education on the age at marriage and total fertility. So women’s education not only has a direct effect in fertility reduction, it also has an indirect effect through the effect on the age at marriage [29].

The examination of the estimated age at completion of childbearing and the average length of the childbearing span by age at marriage showed that women who married younger had a longer childbearing span, which allowed them to produce more children, than their counterparts who married older. This increase of fertility with increased exposure time is consistent with natural fertility [30].

### Contraception

Large fertility decline in the developing world occurred due to a major change in reproductive behaviour of couples in the childbearing ages [31]. More specifically, contraceptive practice has been considered as the interventions of choice for slowing population growth. Bongaat’s stated that the average unweighted net effect of family planning programme is 0.6 births per women which amount to about 30 percent of the observed fertility decline in the developing countries in late 1980s [31, 32].

In less developed countries there was a wide gap in contraceptive prevalence rate between the highest and lowest wealth quintiles [33]. This gap between the rich and poor in the use of contraception has persisted despite general global improvements in socio-economic status and the expansion of family planning services [34]. Health disparities between the rich and poor remain a persistent challenge [35, 36].

The primary reason for the growth in contraceptive prevalence from the 1970s to the late 1980s and 1990s in Latin America, Asia and Africa was that couples who in the earlier period wished to avoid pregnancy but were not using contraceptives were far more likely to be doing so in the more recent period, presumably because of the weakening of obstacles to use that previously prevented them from implementing their fertility preferences [37].

It was argued that significant decrease in fertility was due to social and economic development, and had nothing to do with promotion of family planning. However, Taiwan’s experience shows that, although social and economic development contributed to the decline of fertility after the Second World War, the promotion of a family planning program expedited the process. Moreover, the universal provision of effective and low-cost contraceptives to eligible couples helped the contraceptive practice rate to rise very quickly. These actions enabled couples to control their fertility to the declining level of ideal number of children [38]. Another study in Uganda by Bbaale and Mpuga (2011) found that education, particularly of women, was an important factor in reducing fertility. While the partner’s education was also negatively related to the number of children born, the magnitude was much smaller. There was near universal knowledge of methods of family planning, but very few women used these methods and even fewer the modern methods. Further, access to or use of contraceptives was positively associated with the education of both the woman and her partner [39].

Contraceptive knowledge significantly reduces fertility. Besides, mass media exposure and social networks play important roles in obtaining knowledge of modern contraceptive techniques. Women, who regularly watch TV, listen to the radio, or read newspapers and magazines are more likely to be exposed to contraceptive-related information and hence have more knowledge of contraceptives. Similarly, women who participate in women’s organizations are more likely to obtain contraceptive information through word-of-mouth communication [40].

Ross and Stover (2001) concluded that countries with high social and economic development had high contraceptive prevalence [41]. Studies have also shown that countries in which all couples have easy access to a wide range of contraceptive methods have a more balanced methods mix and higher levels of overall contraceptive prevalence than countries with limited access to various contraceptives [42, 43]. Another study [44] found that the easier the accessibility of contraceptive services to women in a community, the higher the rate of contraceptive use. Total fertility in Vietnam had fallen dramatically due to high rates of contraceptive use and of induced abortion [45].

While women’s education continues to be strongly associated with lower fertility in India, an important feature of India’s current fertility transition is the spread of contraceptive use among uneducated women. Indeed, changes in their fertility are now making the major contribution to the country’s overall fertility decline. The analysis by McNay (2003) suggested that, while many of the expected socio-economic variables play their part, there are also considerable diffusion effects in progress, many of which operate at levels beyond the uneducated women’s own individual circumstances [12]. Another study by Arokiasamy (2009) also concluded that India’s fertility transition is driven by major fertility declines among women who are illiterate. This analysis indicated that illiterate women and their children are the greatest recipients of the benefits of health and socio-economic advancement. The standardised percentages of women without education who received three antenatal care check-ups and whose children received full immunisation are sharply higher for women with two children and less than for those with more than two children. Child mortality reductions for women of lower parities are steeply higher for uneducated women compared with educated women. These cumulative benefits of low fertility, in effect, have speeded up the health improvement and socio-economic advancement of the states [13].

Another study in Indian context found that women who have received family planning messages from health care workers are more likely to use contraceptives as compared to other women. Most of the increase in propensity has been explained by the increase in use rate among women of urban areas and rural women who had access to health facilities. Education continues to have a significant positive influence on contraceptive use however; the differential by educational groups has become much smaller. This change reflects an increase in use rate among women with no education. Therefore, the change in the fertility level of uneducated women is the major factor, which, contributed to a decline in the overall fertility level [46].

Choice of contraceptive depends a lot on the socio-economic and cultural set up of the country. Experience showed that as the contraceptive prevalence increased and people started opting for small family size, use of sterilization increased. The only exception is India where sterilization has dominated the scene right from the beginning, may be more because of programme effort than people choice [47].

### Induced abortion

Induced abortion has long been recognized as one of the principal determinants of fertility levels and is thought to be practiced throughout the world, regardless of its legal status [48]. However, the demographic impact of abortion in developing countries remains uncertain. Without proper estimates of abortion, demographers cannot accurately quantify fertility trends nor can they thoroughly understand the relationships between the determinants of fertility and the fertility level. Data quality is an important consideration in studying the effects of abortion on fertility. Direct measures can be used only where termination of pregnancy is accurately reported. Abortion, however, is generally stigmatized and thus subject to considerable underreporting [49].

Even in the countries with high rates of legal induced abortion, contraceptive use and marital patterns nearly always had a greater impact on fertility levels than abortion. As a rule, extremely high rates of abortion—three or more abortions per woman of childbearing age during the reproductive years—are required for the fertility-inhibiting effect of abortion to rival that of contraceptive use. Nevertheless, the absolute effect of abortion on fertility is often substantial [50].

An analysis of Demographic and Health Survey data provided indirect estimates of the prevalence of abortion in 21 developing countries by rearranging Bongaarts’s proximate determinants model to allow calculation of the index of abortion from other principal proximate determinants of fertility (marriage, contraceptive use and postpartum insusceptibility to pregnancy), average total fecundity and total fertility. On average, abortion appears to have an influence on fertility similar to that of contraceptive use [48].

The prevalence of abortion was high in Vietnam and it was more common among married women aged 20 or older, those with more years of schooling and those with one or two children, as well as among women from the Kinh majority group and the more socio-economically privileged ethnic minorities. It was also found that the abortion rate was higher among women who used a traditional method than among those who used a modern method [51]. Vietnamese provinces exhibited substantial variations in both the rate of abortion and the type of procedures perform [52].

Unsafe abortion is a leading cause of reproductive morbidity and mortality in countries where abortion is illegal or severely restricted. Although Guatemalan law permits induced abortion only to save a woman’s life, many women obtained abortions, often under unsafe conditions and in response to an unintended pregnancy [53]. Another study in India found that a cultural preference for sons may be a factor driving recourse to abortion in India. Rising educational attainment among women may lead to an increase in the demand for abortion [54]. The increase in the level of induced abortion seen in some areas in Philippines reflected the difficulties women experience in obtaining modern contraceptives as a result of social and political constraints that affect care provision [55].

The legal status and the availability of induced abortion are highly variable in Asia [56]. Rising contraceptive use results in reduced abortion incidence in settings where fertility itself is constant. The parallel in abortion and contraception in some countries occurred because increased contraceptive use alone was unable to meet the growing need for fertility regulation in situations where fertility was falling rapidly [57].

### Postpartum insusceptibility

A woman’s exposure to the risk of pregnancy is influenced by postpartum factors: postpartum amenorrhea, which is largely determined by the duration and intensity of breastfeeding and postpartum abstinence, are the two determinants of the duration of postpartum insusceptibility to becoming pregnant. A conception may not always result in a live birth. The outcome of a pregnancy may end in a spontaneous abortion, in an induced abortion, or in a still birth. Postpartum amenorrhea is a biological variable associated with each conception regardless of its outcome [58]. It depends on a number of factors which may vary from woman to woman in a population and for a woman depending on age, marital duration, number of pregnancies, nutritional status, and practice of breast feeding etc [59].

Average birth interval may increase due to longer duration of breastfeeding which in turn reduce fertility particularly in societies where contraceptive use is limited [60, 21, 61, 62, 63, 64, 65, 66]. Similarly, lactation increases the duration of postpartum amenorrhea and that long-term breastfeeding is consistently associated with long periods of postpartum amenorrhea, ovarian inactivity, and reduced fertility [21, 67]. In the absence of breastfeeding the average amenorrheic period may last between one and three months; but when nursing is initiated just after childbirth, the duration of amenorrhea increases systematically with the duration of breastfeeding though at progressively slower rates [62, 68]. Studies also found that the factors responsible for resumption of menstruation are weaning, infant mortality and breastfeeding pattern [69, 70, 71].

## Methods

### Data source

The primary sources of data for this study are the latest available Demographic and Health Surveys (DHS) for six Asian countries. DHS evolved from World Fertility Surveys and Contraceptive Prevalence Surveys implemented in the 1970s and 1980s [72]. Similar to its forerunners, the DHS originally collected comparable population-based data on fertility, contraception, maternal and child health and nutrition in developing countries [72]. The main purpose of the DHS surveys is to provide countries with the data needed to monitor and evaluate population, health and nutrition programmes on a regular basis [73]. Almost 230 nationally representative and internationally comparable DHS household surveys have been conducted in more than 85 countries by national institutions in partnership with ICF Macro International and the United States Agency for International Development (USAID) [72]. In recent times, DHS questionnaires cover a wide range of population and health topics. Moreover, DHS started collecting information regarding specific topics like human immunodeficiency virus (HIV) and acquired immunodeficiency syndrome (AIDS), malaria, and domestic violence through optional modules.

### Selection of the countries

Six Asian countries namely Bangladesh, India, Indonesia, Nepal, Philippines, and Vietnam have been selected for the study purpose. These countries are chosen because their fertility transition has occurred at relatively low socio economic levels, and they are in different stages of fertility transition. Three rounds of DHS surveys for each country (two rounds in Vietnam) are considered for purpose of analysis (Table 1). All DHS surveys are classified into three categories: first period, middle period, and recent period. The survey years for the three periods do not overlap in any of the countries. Also, the inter-survey gap between first and recent period is more or less ten years in five of the sample countries whereas it is five years in case of Vietnam. The data analysis has been done using SPSS version 18.0 and STATA version 10.0 softwares.

**Table 1:** Time description of the Demographic and Health Surveys (DHS) for the study countries.

Country	First period	Middle period	Recent period
Bangladesh (BDHS)	1996–97	1999–2000	2007
India (NFHS)	1992–93	1998–99	2005–06
Indonesia (IDHS)	1998	2003	2007
Nepal (NDHS)	1996	2001	2006
Philippines (NDHS)	1998	2003	2008
Vietnam (VNDHS)	1997	2002	-

## Statistical Analysis

Different statistical analyses are used to fulfil the particular objectives considered for the study and the detailed description of the same are as following:

### Computation of wealth index and operational definition of poor and non-poor

DHS does not collect information on income or expenditure, however, it does include at least 25–30 (and often many more) questions about household characteristics and possessions: materials used for house floors, walls, and roofs; source of water like a stream, open well, or piped system; and presence of durable possessions like a fan, radio, radio receiver, watch, bicycle, or automobile; and other attributes related to economic status [74]. From this information various indicators of household wealth can be constructed [75, 76, 77, 78]. Few authors have shown that the relationship between economic status and fertility obtained with such proxy variables were similar to those measured with indicators such as the expenditures per person [79, 80, 81].

For the study, economic status is measured by computing a ‘wealth index’ using principal component analysis (PCA). Computed wealth index has been standardized by taking the same asset indicators for each of the three time periods. For example, while computing the wealth index for ‘First period’, those variables have been included which are available for all the study countries. Likewise the wealth index for ‘Middle’ and ‘Recent period’ has been constructed. Number of assets under consideration varies across the three time periods but not across the countries. It is observed that over the period of time inclusion of the asset indicators has been increased in DHS surveys which are representative of the economic status of households for that particular time period. So, it is quite obvious that number of assets included in ‘First period’ (11 common assets) are lesser than the ‘Middle period’ (21 common assets) as well as ‘Recent period’ (28 common assets). For enough sample size the computed household wealth index are classified into three equal parts (33.3 percent each). The lowest 33.3 percent is considered as ‘poor’ and the upper 33.3 percent is considered as ‘non-poor’.

### Fertility measurement among poor and non-poor

The fertility data were collected by asking all women of reproductive age (15–49 years) to provide complete birth histories of all children they had given birth to, those who were currently living with them, those who were living away, and those who had died. In addition, the following information was collected for each live birth: name, sex, date of birth, survival status, current age (if alive), and age at death (if dead).

### Estimation of total fertility rate (TFR) among poor and non-poor

Total fertility rate (TFR) is considered as the dependent variable for the purpose of the study. This is an overall summary measure of fertility and is computed from the age-specific fertility rates. Age-specific fertility rates (ASFRs) are computed from the birth history by dividing the number of births to women in a specified age group, during a specified period of time, by the number of woman-years of exposure during the same period. The total fertility rate (TFR) is then obtained by summing the age-specific fertility rates for five-year age groups and multiplying by 5. The total fertility rate therefore indicates the number of children on an average woman would bear from age 15 to age 49 if she experienced the age-specific fertility rates observed during the period for which the total fertility rate is computed. The total fertility rate is estimated by economic status for each country across time periods.

### Estimation of proximate determinant indices among poor and non-poor

Values of each proximate determents of fertility among poor and non-poor are calculated using Bongaart’s proximate determinants of fertility model. Bongaarts demonstrated that 96 percent of the variance in fertility level in a given society is explained by the four principal proximate determinants of regulated fertility viz. the proportion of females married, the prevalence of contraceptive use, the incidence of induced abortion and the fertility inhibiting effect of breast-feeding. In the model, *TFR* is expressed as the product of four indices measuring their fertility inhibiting effect and the total fecundity rate (*TF*). Total fecundity rate is the average number of live births expected among women who during their entire reproductive period remain married, do not use any contraception, do not have any induced abortion and do not breastfeed their children. In such a hypothetical situation the value of varies between 13 and 17 but, the mean value of 15.3 births has been suggested by Bongaarts. The value of most important four indices, based on the proximate determinants explained above, ranges between 0 and 1. When the index is close to 1, the proximate determinant will have a negligible inhibiting effect on fertility, whereas when it takes a value of 0 meaning very low value as zero has not been empirically observed so far, it will have a large inhibiting effect. According to the model:

TFR=Cm*Cc*Ca*Ci*TF

Cm = index of marriage (equals 1 if all women of 15–49 years old are married and 0 in absence of marriage)

Cc = index of contraception (equals 1 in the case of no contraception and 0 if all fecund women use completely effective contraception)

Ca = index of abortion (equals 1 in the case of no induced abortion and 0 if all pregnancies are aborted)

Ci = index of postpartum infecundability (equals 1 in the absence of lactation and postpartum abstinence and 0 if the duration of infecudability is infinite)

TF = Total Fecundity

TFR = Total fertility rate

### Estimation of Cm

In Asian countries, sexual intercourse and child-bearing generally occur only within marriage. Thus, the variables which determine the formation and dissolution of marital unions are very important in determining fertility. Among the various variables related to the formation and dissolution of marital unions, the female age at first marriage and the proportion currently married among women of child-bearing ages are important since they have a major share in determining fertility levels and differentials. The index Cm is calculated as follows:

Cm= ∑{m(i)*g(i)}/∑g(i)

where, *m*(*i*) = *age − specific proportions currently married among females*

*g*(*i*) = *age − specific marital fertility rates*

Thus, the index C*m* is the weighted average of the age-specific proportion of females currently married, with the distribution of *TMFR* by age of women used as weights. This definition takes into account the dependence of the fertility impact of marriage on the age distribution of married women.

In this application, the age-specific marital fertility rate, *g*(*i*), was obtained by dividing the age-specific fertility rate by the proportion of currently married women to total women in each 5-year age group. However, because there were very few married women in the age group 15–19, the calculated *g*(15–19) could be subject to large random errors. To overcome this problem, the marital fertility rate for this age group was computed as: *g*(15–19) = 0.75*g(20–24). The multiplication factor 0.75 is based on Leridon’s observation of associations in marital fertility among age groups [82, 83].

### Estimation of Cc

The index of contraception is inversely related to the prevalence and use effectiveness of contraception. In this application the index Cc is calculated by the following formula:

Cc = 1 – 1.08*u*e

where *u* = *prevalence of current contraceptive use among married women of reproductive age*

e = *average use − effectiveness of contraception*

According to Bongaarts, the average effectiveness is assumed to be 1.0 for sterilization and injectables, 0.95 for IUD, 0.90 for oral pills and 0.70 for all other methods, including condoms (Bongaarts, 1978).

The coefficient 1.08 in this equation is an adjustment factor required because women (couples) do not use contraception if they know or believe that they are sterile. The absence of contraception among such sterile couples implies that contraception is therefore concentrated among non-sterile couples. To take into account the stated concentration of contraception, the variable u, which measures prevalence among all couples, has to be inflated by the sterility correction factor.

### Estimation of *Ca*

The number of births averted per induced abortion is largely independent of the age of woman, however, is strongly related to the practice of contraception following the induced abortion. In Bongaarts model (1983) it is defined as:

Ca=TFR∕{TFR+0.4*(1+u)*TA}

*Index of abortion is calculated from the information of lifetime experience of induced abortions* (*TA*), *contraceptive use* (*u*) *and total fertility rate* (*TFR*).

Although reliable measurements of the prevalence of induced abortion are often lacking, it is well known that induced abortion is practiced in many societies [18]. Data on induced abortion in DHS surveys are limited and country specific. There is no information about timing of induced abortion. Information is available only about the type of (stillbirth/miscarriage/induced abortion) last non-live births which occurred in five years prior to the survey. However, this information does not reveal how many such terminations have taken place in five year period. For example, if a woman undergoes more than one such non-live birth terminations then the available information is only for the last of all such terminations. Using data for last five years estimating total abortion rate *TAR* has serious problems. So, for this study we have assumed such terminations took place in the last two years prior to the survey. Here, the assumption is, it is quite rare to find women experiencing more than one such termination in two years and small number of such cases may not affect our estimation. In particular cases, where the only information of total number of non-live births are available instead of type of non-live births (stillbirth/miscarriage/induced abortion), then all non-live birth pregnancies (last) which occurred within 24 months prior to the survey are tabulated according to mothers age. As it is assumed that, there will not be more than one such pregnancy within two years (restricted to two years), then among these non-live birth terminations, those terminations which occurred before seven months of gestations are assumed to be induced abortions or miscarriages. Further, it is assumed that half of such terminations are induced abortion and remaining half are miscarriages.

### Estimation of *Ci*

Bongaarts estimated that the duration of the postpartum sterility (the period prior to the resumption of ovulation following a birth) is one of the four proximate determinants accounting for most variation in fertility levels among populations. Postpartum sterility varies considerably, not only among individuals, but between different populations. It is more or less coincident with the period of postpartum amenorrhea and is primarily determined by the duration and intensity of breastfeeding.

According to Bongaarts, the index Ci equals the ratio of the total natural fertility rate (*TN*) in the presence and absence of postpartum infecundability. *TN* is estimated as the number of birth intervals that can be fitted between age 15 and the end of childbearing years. Because postpartum infecundability does not affect the duration of the reproductive years, its effect works through modifications of the birth interval. Thus the ratio of natural fertility in the presence and absence of postpartum infecundability is equal to the ratio of the average birth interval without and with postpartum infecundability. In the case of no breastfeeding and no postpartum abstinence, the birth interval, on average, is about 20 months, the sum of 1.5 months of minimum postpartum anovulation, 7.5 months of waiting time to conception, 2 months of time added by spontaneous intrauterine mortality, and 9 months for a full-term pregnancy. In the presence of breastfeeding and postpartum abstinence, the average birth interval is approximately equal to 18.5 months (7.5 + 2 + 9), plus the duration of postpartum infecundability. Hence, Bongaarts proposed the following formula for estimating the index *Ci*.

Ci=20/(18.5+i)

Where, *i is the duration of postpartum infecundability*

### Decomposition of change in fertility over time

Any change in the level of fertility of a population is necessarily caused by a change in one or more of the proximate determinants. In order to quantify the contribution made by each proximate determinant, a decomposition procedure is applied to evaluate the impact of the four principal factors on fertility between first and recent survey period by economic status for each study countries. The decomposition of the proportional change in the *TFR* into the components requires the calculation of P values (defined as proportional change in TFR due to changes in different proximate variables) from the indexes *Cm*, *Cc*, *Ca*, and *I is the interaction factor* [84].

Let 1992 and 1998 be the first and recent years of the time period for which decomposition is done. Then, with a change in the *TFR* from *TFR*_1992_ in the year 1992 to *TFR*_1998_ in the year 1998 and with simultaneous changes in the indexes from *Cm*_1992_ to *Cm*_1998_, from *Cc*_1992_ to *Cc*_1998_, from *Ca*_1992_ to *Ca*_1998_, from *Ci*_1992_ to *Ci*_1998_ and from *TF*_1992_ to *TF*_1998_ between the years 1992 and 1998, the ratio *TFR*_1998_/*TFR*_1992_ can be expressed as:

TFR1998/TFR1992=(Cm1998/Cm1992)*(Cc1998/Cc1992)*(Ca1998/Ca1992)*(Ci1998/Ci1992)*(TF1998/TF1992)

Proportional change in TFR between the years 1992 and 1998: Pf = TFR1998/TFR1992–1

Proportional change in TFR due to a change in the index of marriage between the years 1992 and 1998: Pm = Cm1998/Cm1992–1

Proportional change in TFR due to a change in the index of contraception between the years 1992 and 1998: Pc = Cc1998/Cc1992–1

Proportional change in TFR due to a change in the index of induced abortion between the years 1992 and 1998: Proportional change in TFR due to a change in the index of postpartum infecundability between the years 1992 and 1998: Pi = Ci1998/Ci1992–1

Therefore, Pf = Pm + Pc + Pa + Pi + I

Where, *I is the interaction factor*

Thus, the proportional change in TFR between 1992 and 1998 is “equal to the sum of the proportional fertility changes due to the different proximate determinants plus an interaction term” [85]. Decomposition of the change in total fertility rate is estimated for poor and non-poor separately for countries studied and further, distribution of percentage change in TFR and absolute change in TFR are also tabulated.

## Results

### Levels, trends and patterns of fertility among poor and non-poor

The TFR can be interpreted as the number of children a woman would have throughout her life time, if she experiences the same level and pattern of fertility as measured at the time of survey [86]. The TFR is a synthetic cohort measure and thus, does not represent the experience of any real cohort. So, for the study, we have estimated age specific fertility rates (ASFR) and total fertility rates (TFR) for the respective study countries. Further, we have also estimated age specific fertility rates and total fertility rates for poor and non-poor using birth history data. The levels, trends and patterns of fertility are presented in Table 2. Overall trends in TFR indicated that, in the past couple of years all the countries have experienced fertility decline though the level varied. Total fertility rate had declined (from first survey period to recent period) to less than three children per woman in Bangladesh (2.70), India (2.68) and Indonesia (2.60). Among the study countries, Nepal (3.10) and Philippines (3.30) still had total fertility rate more than three children per woman according to 2006 and 2008 survey respectively. Vietnam was the exception with critically low level of fertility (1.87 in 2002). Hence, it can be assumed that, the above mentioned first three countries continued to be in the near replacement level fertility whereas; last two countries were in the transitional phase.

**Table 2:** Estimated total fertility rates (TFR) by economic status in selected Asian countries according to DHS data.

Country/Survey	Total fertility rates (TFR)		
	Poor	Non-poor	Total
Bangladesh			
1996–97*	3.48	2.83	3.27
1999–2000**	3.92	2.76	3.31
2007***	2.89	2.45	2.70
India			
1992–93*	3.71	2.92	3.39
1998–99**	3.17	2.54	2.85
2005–06***	3.42	1.82	2.68
Indonesia			
1997*	3.15	2.59	2.78
2003**	2.69	2.50	2.60
2007***	2.68	2.59	2.60
Nepal			
1996*	5.13	3.87	4.64
2001**	4.83	2.97	4.10
2006***	4.31	2.18	3.10
Philippines			
1998*	5.90	2.62	3.73
2003**	5.48	2.44	3.50
2008***	4.82	2.22	3.30
Vietnam			
1997*	2.67	2.00	2.33
2002**	2.02	1.88	1.87

Note:

*refers to first period,

**refers to middle period,

***refers to recent period

Bifurcation of total fertility rate by economic status presents a far clearer picture about fertility behavior among poor and non-poor women in six Asian countries. Results (Table 2) indicate that fertility level differed within and across the countries irrespective of economic status. Interestingly, the pace of fertility decline was pretty faster among poor women in Bangladesh compared to their non-poor counterparts. In the first survey period (1996–97), on an average, one poor woman had 3.48 children which peculiarly rose up to 3.92 children in 1999–2000 and again declined to 2.89 children in 2007, whereas, in case of non-poor women the TFR declined consistently from 2.83 to 2.76 from 1996–97 to 1999–2000 time period and further declined from 2.76 to 2.45 during 1999–2000 to 2007 time period. Hence, the gap between poor and non-poor women in terms of total fertility rate had narrowed down gradually over the period of time.

In India, fertility level was comparatively higher among poor women than non-poor women. However, over the period of time fertility had shown a declining trend among both groups of women. During the fifteen years time period total fertility rate among poor had declined from 3.71 in 1992–93 to 3.42 in 2005–06. On the other hand, pace of fertility decrease was comparatively faster among non-poor women in India. According to 1992–93 data, on an average one non-poor woman had almost 3 children during her reproductive span which had declined to 2.54 in 1998–99 and finally it was below the replacement level fertility (1.82) in 2005–06. Data also revealed that, fertility decline among non-poor women in India was much consistent compared to their poor counterparts (Table 2).

Interesting result (Table 2) was noticed in case of Indonesia, as its pace of fertility decline was quite fast among poor women compared to their non-poor counterparts. For example, total fertility rate had declined from 3.15 in 1997 to 2.68 in 2007 however; there was no change in fertility level among non-poor women during the ten years (1997 to 2007) time period. In 1997, TFR among non-poor Indonesian women was 2.59 which have remained the same in 2007. Hence, it was clear from the analysis that fertility decline was much of a reality for poor women and fertility behaviour had changed progressively among that particular group of the society compared to their non-poor counterparts in Indonesia.

Fertility level was quite high among poor women in Nepal (Table 2). On an average one poor woman had more than 5 children in her reproductive span (5.13 in 1996) however; it declined to 4.31 during ten years time period (1996 to 2006). On the other hand, fertility level was comparatively at a much lower level among non-poor women than their counterparts. Total fertility rate among non-poor women was 3.87 in 1996 which had gradually declined and according to 2006 data non-poor women have achieved replacement level fertility (2.18). Thus, there was a sharp gap between poor and non-poor women in terms of fertility level and also the fertility behavior was quite unusual especially among poor women.

Among all the study countries, the fertility scenario was entirely different in Philippines and there was a sharp contrast among poor and non-poor women (Table 2). A poor woman had experienced approximately 6 children during her reproductive span in 1998 and the corresponding figure for the non-poor women was 2.62 which was much lower than poor and the gap was quite appreciable. Over the period (1998 to 2008), fertility had declined slowly and in 2008, fertility among poor and non-poor women was 4.82 and 2.22 respectively. Therefore, even-tough non-poor women were about to achieve the replacement level fertility however; there was substantial room available for poor women to reduce their fertility level.

Vietnam was one such country where the fertility was well controlled by both poor and non-poor women (Table 2). Results demonstrated that, the country had achieved replacement level fertility much earlier. Total fertility rate among poor women was 2.67 in 1997 and further it was below replacement level (2.02) in 2002. On the other hand, non-poor women have achieved below replacement level fertility (2.00) in 1997 while according to 2002 data, fertility was at critically low level (1.88).

Across country comparison indicate that, the six Asian countries had considerable diversity with regards to TFR, in poor women aged 15–49, TFR ranged from 4.82 in Philippines to 2.02 in Vietnam according to most recent survey. Further, between the highest and lowest fertility mentioned earlier, total fertility rate among other Asian countries was 4.31 in Nepal, 3.42 in India, 2.89 in Bangladesh and 2.68 in Indonesia. It signifies that, fertility level differed among poor women from one country to another and women have experienced different kind of fertility behavior and child bearing practices. However, compared to poor women, non-poor women’s fertility level was lower and it was more or less at the same level in Indonesia (2.59 in 2007), Bangladesh (2.45 in 2007), Philippines (2.22 in 2008) and Nepal (2.18 in 2006). Thus, they were either very close to near replacement level or had already achieved replacement level fertility according to the recent surveys in respective countries. Interestingly, non-poor women in India (1.82) have achieved below replacement level fertility in 2005–06 and out of six countries both poor (2.02) and non-poor (1.88) women in Vietnam reached below replacement level fertility much earlier in 2002 (Table 2).

### Proximate determinants of fertility by economic status

In order to improve the understanding of fertility behavior, its variations among different sub-groups and underlying causes of the variations, it is necessary to analyze the mechanisms through which proximate determinants influence fertility. Kingsley Davis and Judith Blake were the first to identify the factors which influence fertility directly with the help of eleven “intermediate fertility variables” [87]. Further it was modified by John Bongaarts and he even identified eight direct determinants of fertility which he called the “proximate determinants of fertility”. According to John Bongaarts and Robert Potter, the proximate determinants of fertility are the biological and behavioural factors through which social, economic and environmental variables affect fertility [62].

According to Bongaarts’s (1982) framework, all important variations in fertility are captured by variation in the proximate determinants of fertility. Bongaarts demonstrated that almost 96 percent variability in fertility within a society can be explained by the above mentioned first four variables and the effect of the last four variables are almost negligible. Therefore, if we have good enough individual-level data on marriage, contraceptive use, abortion (not mandatory), post-partum amenorrhoea and the other proximate determinants, all variations in individual-level fertility can be captured. Evidence suggests that, DHS calendar data are among the most detailed data which can capture the measurable variation in fertility.

Thus, in this paper, we have quantified the proximate determinant indices to capture the relative contribution of each proximate determinant viz. marriage, contraception, postpartum infecundability and abortion on fertility reduction among two distinct group of population i.e. poor and non-poor. Further, we have determined whether the observed total fertility rates based on the DHS data are consistent with the predicted rates based on the Bongaarts proximate determinant model. We then assessed the values of the proximate determinants and their contributions to fertility changes among poor and non-poor.

### Proximate determinant Indices by economic status

**Index of marriage** (*Cm*). Early marriage has direct implications for exposure to childbearing that can lead to higher cumulative fertility over the lifespan [88]. To assess the impact of marriage on fertility, we compared the marriage index (*Cm*) across economic groups for selected study countries (Table 3). The estimated values for *Cm* showed that marriage timing was particularly important as a determinant of overall fertility among non-poor women compared to poor women particularly in Philippines followed by Vietnam, India, Indonesia, Nepal and Bangladesh. The fertility depressing impact had increased from 0.349 in 1998 to 0.271 in 2008 in Philippines; however, the corresponding figure for poor was relatively high, nonetheless; trends showed that gradually it had declined from 0.646 in 1998 to 0.534 in 2008.

**Table 3:** Estimated total fertility rate (TFR), adjusted total marital fertility rate (TMFR) and index of *Cm* by economic status in selected Asian countries according to DHS data.

Country/Survey	Poor			Non-poor		
	TFR	TMFR	*Cm*	TFR	TMFR	*Cm*
Bangladesh						
1996–97*	3.48	4.04	0.861	2.83	3.29	0.861
1999–2000**	3.92	4.75	0.825	2.76	3.25	0.850
2007***	2.89	3.36	0.857	2.45	2.93	0.835
India						
1992–93*	3.71	4.43	0.838	2.92	4.44	0.657
1998–99**	3.17	3.88	0.818	2.54	4.01	0.632
2005–06***	3.42	4.19	0.814	1.82	3.39	0.535
Indonesia						
1997*	3.15	4.50	0.701	2.59	3.81	0.679
2003**	2.69	4.11	0.655	2.50	3.83	0.653
2007***	2.68	4.09	0.655	2.59	3.82	0.676
Nepal						
1996*	5.13	4.30	0.823	3.87	4.71	0.821
2001**	4.83	5.94	0.812	2.97	3.73	0.796
2006***	4.31	5.32	0.809	2.18	3.16	0.689
Philippines						
1998*	5.90	9.14	0.646	2.62	7.50	0.349
2003**	5.48	8.97	0.611	2.44	6.66	0.367
2008***	4.82	9.03	0.534	2.22	8.18	0.271
Vietnam						
1997*	2.67	4.92	0.543	2.00	3.76	0.532
2002**	2.02	4.18	0.483	1.88	3.70	0.507

Note:

*refers to first period,

**refers to middle period,

***refers to recent period

Vietnam and Indonesia exhibited that the impact of marriage had increased among poor (0.543 in 1997 to 0.483 in 2002 and 0.701 in 1996 to 0.655 in 2007 respectively) compared to non-poor (0.532 in 1997 to 0.507 in 2002 and 0.679 in 1996 to 0.676 in 2007 respectively) over time. Index value of *Cm* also revealed that inhibiting effect of marriage on fertility had a growing impact among non-poor women in India (0.657 in 1992–93 to 0.535 in 2005–06), poor women in Indonesia (0.701 in 1997 to 0.655 in 2007) and non-poor women in Nepal (0.821 in 1996 to 0.689 to 2006). Although, the impact of marriage on fertility was comparatively less among both poor and non-poor women in Bangladesh, and poor women in India and Nepal, nevertheless, the consistent lower index value of *Cm* indicated that impact of marriage had gradually increased in the above stated countries, however in a sloth pace.

**Index of contraception** (Cc). Trends in index of contraception (*Cc*) by economic status are presented in Table 4. Results illustrated that contraception had the most effective influence on fertility reduction among both poor and non-poor women although the level varied within and across the countries over the period of time. Interestingly, the fertility inhibiting effect of contraception was highest among poor women in Vietnam and it had increased over time (0.333 in 1997 to 0.284 in 2002) compared to non-poor (0.260 in 1997 to 0.301 in 2002). Although the fertility inhibiting effect on fertility due to contraception was higher among non-poor women compared to poor women in rest of the countries, however; trends indicated the effect of contraception had increased gradually among both group of women at varied level over time. For example, the inhibiting effect had increased rapidly among poor than non-poor (0.063 vs. 0.025 respectively) women in Indonesia from 1997 to 2007 time period. In India, the index value of *Cc* had decreased from 0.456 in 1992–93 to 0.346 in 2005–06 among non-poor however the corresponding figure for poor women was 0.675 to 0.526 during the same period. The gap between poor and non-poor women in terms of estimated value of *Cc* had narrowed down in Bangladesh because of the comparatively faster increase in fertility inhibiting effect of contraception among poor (0.089 point increase among poor and 0.021 among non-poor) during 1996/97 to 2007 time period. The estimated lower value of *Cc* confirmed the increasing inhibiting effect on fertility in Nepal and Philippines also. Despite the differences in pace of decline within and across the study countries, inhibiting effect of contraception on fertility emerged as a very important factor irrespective of economic status.

**Table 4:** Estimated index of by economic status in selected Asian countries according to DHS data.

Country/Survey	*Cc*	
	Poor	Non-poor
Bangladesh		
1996–97*	0.567	0.486
1999–2000**	0.534	0.462
2007***	0.478	0.465
India		
1992–93*	0.675	0.456
1998–99**	0.618	0.398
2005–06***	0.526	0.346
Indonesia		
1997*	0.500	0.391
2003**	0.466	0.366
2007***	0.437	0.366
Nepal		
1996*	0.794	0.591
2001**	0.704	0.456
2006***	0.595	0.438
Philippines		
1998*	0.654	0.524
2003**	0.619	0.514
2008***	0.584	0.515
Vietnam		
1997*	0.333	0.260
2002**	0.284	0.301

Note:

*refers to first period,

**refers to middle period,

***refers to recent period

**Index of abortion** (Ca). It was already recommended by Bongaarts (1983) that the index of abortion may be assumed as one due to lack of data. However, we have tried to estimate the index of abortion (*Ca*) with the information available in DHS surveys for respective countries. The estimated values of index of abortion by economic status across Asian countries are presented in Table 5. According to our estimated values of *Ca*, it was found that in a broader sense induced abortion had almost negligible inhibiting effect on fertility among both poor and non-poor women across the countries and over time. Nonetheless, in case of Vietnam, the declining value of *Ca* indicated that the inhibiting effect of abortion might have a little influence on fertility reduction among non-poor (0.884 in 1997 to 0.824 in 2002) and poor women (0.909 in 1997 to 0.890 in 2002).

**Table 5:** Estimated index of Ca by economic status in selected Asian countries according to DHS data.

Country/Survey	Total abortion rate (TAR)		*Ca*	
	Poor	Non-poor	Poor	Non-poor
Bangladesh				
1996–97*	0.172	0.325	0.972	0.934
1999–2000**	0.202	0.460	0.970	0.904
2007***	0.150	0.355	0.969	0.916
India				
1992–93*	0.028	0.084	0.996	0.982
1998–99**	0.024	0.108	0.996	0.973
2005–06***	0.101	0.200	0.983	0.931
Indonesia				
1997*	0.002	0.029	1.000	0.993
2003**	0.114	0.221	0.975	0.945
2007***	0.162	0.246	0.964	0.941
Nepal				
1996*	0.166	0.209	0.985	0.971
2001**	0.174	0.168	0.982	0.966
2006***	0.275	0.271	0.966	0.927
Philippines				
1998*	0.005	0.001	1.000	1.000
2003**	0.310	0.184	0.969	0.956
2008***	0.003	0.001	1.000	1.000
Vietnam				
1997*	0.394	0.364	0.909	0.884
2002**	0.356	0.563	0.890	0.824

Note:

*refers to first period,

**refers to middle period,

***refers to recent period

**Index of postpartum infecundity** (Ci). The magnitude of postpartum infecundity by economic status is presented in Table 6. The lower values of *Ci* among poor compared to their non-poor counterparts indicated that the fertility inhibiting effect due to postpartum infecundity has increased among poor women. Among the study countries the estimated values of *Ci* were lowest among both poor (0.637 in 1996 to 0.633 in 2006) and non-poor women (0.733 in 1996 to 0.699 in 2006) in Nepal. Another interesting result was found in Vietnam. Among the poor women in Vietnam, the fertility inhibiting effect due to postpartum infecundity has decreased (0.712 in 1997 to 0.816 to 2002) over time however; among non-poor it has slightly increased (0.772 in 1997 to 0.746 in 2002).

**Table 6:** Estimated index of *Ci* by economic status in selected Asian countries according to DHS data.

Country/Survey	Postpartum insusceptibility		*Ci*	
	Poor	Non-poor	Poor	Non-poor
Bangladesh				
1996–97*	10.4	3.0	0.692	0.813
1999–2000**	10.5	7.6	0.690	0.766
2007***	6.9	5.1	0.787	0.847
India				
1992–93*	11.3	6.0	0.671	0.803
1998–99**	12.5	5.8	0.645	0.823
2005–06***	10.0	4.8	0.702	0.858
Indonesia				
1997*	7.9	4.4	0.758	0.873
2003**	6.0	3.3	0.803	0.917
2007***	5.5	2.9	0.833	0.935
Nepal				
1996*	12.9	8.8	0.637	0.733
2001**	13.4	10.0	0.627	0.702
2006***	13.1	10.1	0.633	0.699
Philippines				
1998*	8.7	3.8	0.735	0.897
2003**	8.2	5.3	0.749	0.840
2008***	7.0	3.5	0.784	0.909
Vietnam				
1997*	9.6	7.4	0.712	0.772
2002**	6.0	8.3	0.816	0.746

Note:

*refers to first period,

**refers to middle period,

***refers to recent period

### Contributions to the change in TFR

Any change in the level of fertility of a population is necessarily caused by a change in one or more of the proximate determinants. In order to quantify the contribution made by each proximate determinant, a decomposition procedure was applied to evaluate the impact of the four principal factors on fertility between first to recent survey for respective countries. The decomposition of the proportional change in TFR due to changes in different proximate variables has been estimated from the indexes Cm, Cc, Ca and Ci and it is presented in Table 7.

**Table 7:** Decomposition of the change in total fertility rate (TFR) in Asian countries from first period to recent period.

Factors responsible for fertility change												
	Bangladesh		India		Indonesia		Nepal		Philippines		Vietnam	
	(1996–97 to 2007)		(1992–93 to 2005–06)		(1997 to 2007)		(1996 to 2006)		(1998 to 2008)		(1997 to 2002)	
	Poor	Non poor	Poor	Non poor	Poor	Non Poor	Poor	Non Poor	Poor	Non poor	Poor	Non poor
	**Percentage of change in TFR**											
Proportion of women married	-0.441	-3.026	-2.874	-18.593	-648	-0.534	-1.592	-16.070	-17.299	-22.343	-10.978	-4.779
Contraceptive practice	-15.661	-4.340	-22.034	-24.063	-12.558	-627	-25.088	-25.827	-10.588	-1.789	-14.602	15.884
Induced abortion	-0.338	-1.877	-1.321	-5.206	-3.584	-5.187	-1.928	-4.462	0.011	-0.004	-2.041	-6.757
Duration of postpartum infecundity	13.780	237	4.561	6.867	10.000	7.009	-0.633	-4.545	6.667	1.364	14.694	-3.358
Interaction	-1294	-8.422	13.716	3.259	-2.330	4.945	13.339	7.106	2.905	7.476	-11.418	-7.240
Total	-16.954	-13.428	-7.951	-37.736	-14.921	-0.193	-15.902	-43.798	-18.305	-15.296	-24.345	-450
	**Distribution of percentage of change in TFR**											
Proportion of women married	-2.602	-22.533	-3342	-49.270	-43.214	-27682	-10.011	-36.690	-94.502	-146.065	-45.093	-7672
Contraceptive practice	-92.374	-32.324	-277.108	-63.767	-84.168	-3329.050	-157.764	-58.969	-57.845	-11.697	-59.979	254.144
Induced abortion	-1.992	-13.978	-16.615	-13.796	-24.022	-2686.897	-12.125	-10.187	0.059	-0.028	-8.382	-108.105
Duration of postpartum infecundity	81.276	31.557	57.365	18.197	67.021	3630.841	-3.980	-10.378	3620	8.915	60.358	-53.731
Interaction	-84.308	-62.722	172.501	8.635	-15.617	2561.588	83.880	1424	15.868	48.875	-46.903	-115.835
Total	-100	-100	-100	-100	-100	-100	-100	-100	-100	-100	-100	-100
	**Absolute change in TFR**											
Proportion of women married	-0.015	-0.086	-0.107	-0.542	-0.203	-0.014	-0.082	-0.622	-1.021	-0.584	-0.293	-0.096
Contraceptive practice	-0.545	-0.123	-0.817	-0.701	-0.396	-0.166	-1.286	-1.000	-0.625	-0.047	-0.390	0.318
Induced abortion	-0.012	-0.053	-0.049	-0.152	-0.113	-0.134	-0.099	-0.173	0.001	0.000	-0.054	-0.135
Duration of postpartum infecundity	0.480	0.120	0.169	0.200	0.315	0.182	-0.032	-0.176	0.393	0.036	0.392	-0.067
Interaction	-0.497	-0.238	0.509	0.095	-0.073	0.128	0.684	0.275	0.171	0.196	-0.305	-0.145
Total	-0.590	-0.380	-0.295	-1.100	-0.470	-0.005	-0.815	-1.695	-1.080	-0.400	-0.650	-0.125

Results demonstrated that the highest fertility decline occurred among non-poor women in Nepal (44 percent) during 1996 to 2006 time period which can be attributed to a 26 percent decline due to increasing contraceptive practice, 16 percent resulting from a decrease in the proportion of women married, 5 percent fall due to the prolongation of the duration of postpartum infecundability and 4 percent due to an increase in the practice of induced abortions. Compared to non-poor women, total 16 percent fertility had declined among poor women in Nepal. Out of the 16 percent fertility decline, a bulk of fertility (25 percent) had reduced because of increasing contraceptive practices. Other factors i.e. non-marriage (2 percent), induced abortion (2 percent), postpartum infecundity (1 percent) had a minimal contribution on fertility decline in last 10 years in Nepal.

In past 13 years, about 38 percent fertility had declined among non-poor women in India however; the corresponding figure for the poor women was 8 percent. The decomposition analysis revealed that 22 percent and 24 percent fertility had declined due to increasing contraceptive practices among poor and non-poor women respectively. Second most important fertility inhibiting factor among non-poor was decrease in the proportion of women married (19 percent fertility decline), however; its impact was very minimal (3 percent fertility decline) on poor women in India. The relative contribution of induced abortion on fertility decline was about 5 percent for non-poor women and only 1 percent among poor in India.

Interestingly, substantial fertility had declined among poor women compared to their non-poor counterparts in Vietnam (24 percent against 6 percent), Philippines (18 percent against 15 percent), Bangladesh (17 percent against 13 percent) and Indonesia (15 percent against 1 percent). It was also observed that, majority of the fertility decline in the above stated countries was primarily because of massive increase in contraceptive practices and it was higher, to a great extent, among poor than non-poor women in Bangladesh (16 percent vs. 4 percent fertility decline), Indonesia (13 percent vs. 6 percent fertility decline) and Philippines (11 percent vs. 2 percent fertility decline). The relative contribution of proportion of non-marriage was the second most important fertility inhibiting factor among poor women in Philippines (17 percent fertility decline) and Indonesia (6 percent fertility decline). Fertility decline among poor in Vietnam was largely attributed to increasing contraceptive practices (15 percent fertility decline) followed by proportion of non-marriage (11 percent fertility decline) and a small amount of contribution of induced abortions (2 percent fertility decline). However, fertility decline among non-poor women in Indonesia can be attributed to an increase in practice of contraceptive use and induced abortion (6 percent and 5 percent fertility decline respectively). In case of Philippines, the sole reason for fertility decline was contribution of non-marriage (22 percent fertility decline). Interestingly, fertility decline among non-poor women in Vietnam had occurred mostly because of increase in the practice of induced abortion (7 percent fertility decline) followed by increase in proportion of non-marriage (5 percent fertility decline) and the prolonged duration of postpartum infecundability (3 percent fertility decline).

## Discussion

Undoubtedly, fertility transition is well underway in the six study countries. Fifty years back fertility of these countries was at a different paradigm and it had started declining in each of the study countries at different point of time. Overall fertility decline has been a continuous process in these countries along with the substantial fertility decline among two distinct groups i.e. poor and non-poor. However, the pace of decline varied within and across the countries. Non-poor women started controlling their fertility much earlier and there was a time lag in case of poor women. In some countries like Nepal, Philippines and India fertility level was still far from satisfactory particularly among poor women. In contrast, fertility decline has been overwhelming among the poor in Bangladesh, Indonesia, Philippines and Vietnam. It was noteworthy that, out of the six countries both poor and non-poor women in Vietnam have achieved the replacement levels of fertility much before the other countries.

Thus, the results revealed that there has been a gradual change in fertility behaviour even among the disadvantaged section of the society. The changing behavior among poor women may be due to the perceived benefits of small family size. Therefore, it may be assumed that, fertility behavior of non-poor women will be diffused gradually among poor women and their fertility will continue to decline until and unless they achieve the replacement level of fertility. However; it is imperative to study the determining factors for the changing fertility behaviour among sub-group of population in a particular society.

While analyzing the determining factors for fertility decline, it was found that fertility has declined among both poor and non-poor women during ten years or more (except Vietnam) time period due to the changes in three principal proximate determinants i.e. increasing proportion of women currently practicing contraception, decreasing proportion of women currently married and an increasing rate of induced abortions with the exception of postpartum infecundibility. The trends in all three factors confirmed that the fertility inhibiting effect has increased among both poor and non-poor women however; the level varied within and across the countries. The increasing index value of *Ci* indicated the decreasing inhibiting effect of duration of breastfeeding and menstrual regulations on fertility.

There was substantial fertility decline, especially among poor, across all the countries over the period of time. It was quite clear from the analysis that out of four proximate determinants of fertility, contraception had the largest effect and it was the sole reason for fertility decline particularly among poor women in Nepal followed by India, Bangladesh, Vietnam, Indonesia and Philippines between first to recent survey period. However, the magnitude of inhibiting effect of contraceptives varied among poor women in study countries. Nevertheless, the importance of contraceptive practices for fertility regulation among poor should not be overlooked as far as fertility decline in Asian countries is concerned.

The second most important fertility inhibiting factor was effect of marriage. Earlier it was observed that marriage was almost universal especially among poor and therefore the relative contribution of marriage on fertility reduction was almost negligible. However, the decreasing estimated values of *Cm* indicated that gradually the effect of marriage had increased and the marriage pattern had changed gradually among poor. Numerous studies using World Fertility Survey data have shown that the proportion of women married in a population is a major determinant of the level of natural fertility in that population [89]. We also found that marriage has a significant effect on fertility decline especially among poor in Philippines, Vietnam and Indonesia and among non-poor in Philippines, India, Nepal, Vietnam and Bangladesh.

Abortion had a limited fertility inhibiting effect compared to other two proximate determinants and it was the third important proximate determinant for explaining the fertility decline. Analysis showed practice of induced abortion varied across the countries by economic status. For example, fertility inhibiting effect due to induced abortion was higher among non-poor women in Vietnam (7 percent) followed by non-poor women in India (5 percent), Indonesia (5 percent) and Nepal (4 percent). On the other hand, relative contribution of induced abortion on fertility was relatively less among poor women and it brought about 4 percent fertility decline in Indonesia, 2 percent decline in Vietnam, 2 percent decline in Nepal and 1 percent in India. The survey data reflected increase in the number of reported induced abortion by both poor and non-poor women, however; the abortion data were still under reported.

Our analysis also showed that breast feeding, operating through postpartum amenorrhea, had a lesser amount of impact on fertility reduction and the impact was primarily in case of non-poor women in Nepal (5 percent fertility decline) and non-poor women in Vietnam (3 percent fertility decline). Otherwise it acted as a fertility enhancing factor irrespective of economic status across study countries.

We conclude, out of the four proximate determinants, it was the adoption of contraception that contributed most to fertility changes especially among poor in a given time frame. Of the total decline in TFR across the countries, increased contraceptive use was the single reason for the explanation of fertility reduction in most of the cases. Increased proportions unmarried also contributed in fertility decline. Besides, while marriage had also contributed in reducing the potential level of fertility, however; it has been the increased demand for fertility control, expressed through greater use of contraception. Moreover, abortion has been another emerging factor in the changes in fertility level over time.

## Limitations

The results of the study should be interpreted considering the few limitations detailed below:

1. In the absence of data on direct income and expenditure; this study defined economic status on the basis of household ownership and consumer assets.
2. Despite the usefulness of birth histories, DHS surveys are subject to few data quality problems [90, 91, 92, 93, 94, 95]. One of the most commonly mentioned problems of DHS birth histories is the displacement of births [90, 95]. Omission of births is another important constraint for birth histories [91, 96]. We have also found there are under-reporting of births particularly among poor in India and Bangladesh.
3. It is well known that induced abortion is practiced in many societies [18] however; reliable information on induced abortion is often lacking and quite limited in DHS surveys. Sometimes it is only country specific and the quality of data also varies across the countries.
4. We have found that interaction factor arrived while decomposing the TFR in the decomposition analysis varies across the studied countries and among both poor and non-poor women from first to the recent survey period. Such interaction factors may be due to some ‘unexplained’ variables or other proximate determinants of fertility like potential impact of induced abortion, coital frequency, temporary separations, foetal mortality and secondary sterility which were not captured in the model [97].
5. Finally, it was assumed that the value of total fecundity is same among both poor and non-poor. However, it could differ due to several biological or social factors which lead to higher or lower level of TF.
